# TarNet: An Evidence-Based Database for Natural Medicine Research

**DOI:** 10.1371/journal.pone.0157222

**Published:** 2016-06-23

**Authors:** Ruifeng Hu, Guomin Ren, Guibo Sun, Xiaobo Sun

**Affiliations:** 1 Beijing Key Laboratory of Innovative Drug Discovery of Traditional Chinese Medicine (Natural Medicine) and Translational Medicine, Institute of Medicinal Plant Development, Peking Union Medical College and Chinese Academy of Medical Sciences, Beijing, China; 2 Key Laboratory of Bioactive Substances and Resource Utilization of Chinese Herbal Medicine, Ministry of Education, Beijing, China; 3 Zhongguancun Open Laboratory of the Research and Development of Natural Medicine and Health Products, Beijing, China; Shool of Pharmaceutical Sciences, Sun Yet-Sen University, 132 Easy Cycle at University City, CHINA

## Abstract

**Background:**

Complex diseases seriously threaten human health. Drug discovery approaches based on “single genes, single drugs, and single targets” are limited in targeting complex diseases. The development of new multicomponent drugs for complex diseases is imperative, and the establishment of a suitable solution for drug group-target protein network analysis is a key scientific problem that must be addressed. Herbal medicines have formed the basis of sophisticated systems of traditional medicine and have given rise to some key drugs that remain in use today. The search for new molecules is currently taking a different route, whereby scientific principles of ethnobotany and ethnopharmacognosy are being used by chemists in the discovery of different sources and classes of compounds.

**Results:**

In this study, we developed TarNet, a manually curated database and platform of traditional medicinal plants with natural compounds that includes potential bio-target information. We gathered information on proteins that are related to or affected by medicinal plant ingredients and data on protein–protein interactions (PPIs). TarNet includes in-depth information on both plant–compound–protein relationships and PPIs. Additionally, TarNet can provide researchers with network construction analyses of biological pathways and protein–protein interactions (PPIs) associated with specific diseases. Researchers can upload a gene or protein list mapped to our PPI database that has been manually curated to generate relevant networks. Multiple functions are accessible for network topological calculations, subnetwork analyses, pathway analyses, and compound–protein relationships.

**Conclusions:**

TarNet will serve as a useful analytical tool that will provide information on medicinal plant compound-affected proteins (potential targets) and system-level analyses for systems biology and network pharmacology researchers. TarNet is freely available at http://www.herbbol.org:8001/tarnet, and detailed tutorials on the program are also available.

## Background

The “single gene, single drug, single target” drug discovery approach was recently found to be limited when applied to the treatment of complex diseases [1–3]. Network pharmacology has become central to the development of innovative drug discoveries for complex diseases [4–7]. The search for new molecules is currently taking a different approach, whereby the principles of ethnobotany and ethnopharmacognosy are being used by chemists to identify sources and classes of compounds. Medicinal plants have provided humans with medicines since the medieval time, which was about 60,000 years ago, and such medicines typically have effects at the system levels with unclear mechanisms [3, 8–10]. Herbal medicines have formed the basis of sophisticated systems of traditional medicine, resulting in the development of some key drugs that remain in use today. However, the pharmacological mechanisms of these systems remain unclear despite their curative effects.

Traditional approaches used to examine individual genes and proteins are limited in addressing complex diseases [4]. Health and disease-related protein–protein interaction (PPI) network analyses are essential to the resolution of systems biology problems [11]. Furthermore, an increasing body of evidence suggests a relationship between the topological properties and biological functions of protein nodes in networks [4, 11, 12]. Systematic investigations of disease-specific proteins in the human PPI network may provide important biological information needed to uncover the molecular mechanisms of complex diseases [13–18].

To bridge the gap between traditional herbal medicine systems and modern molecular biology as well as to further network pharmacological research, we developed TarNet, a manually curated database and platform of traditional medicinal plants with natural compounds that includes potential bio-target information. TarNet integrates a server-side database of human PPI information, traditional medicinal plant information, natural compound and potential bio-target information, as well as client-side network visualizations. All data included in TarNet were collected from databases or were text-mined and manually curated from publications.

TarNet is free and open to all users without login requirements at http://www.herbbol.org:8001/tarnet.

## Construction and Implementation

### Data collection

**Fig 1:**
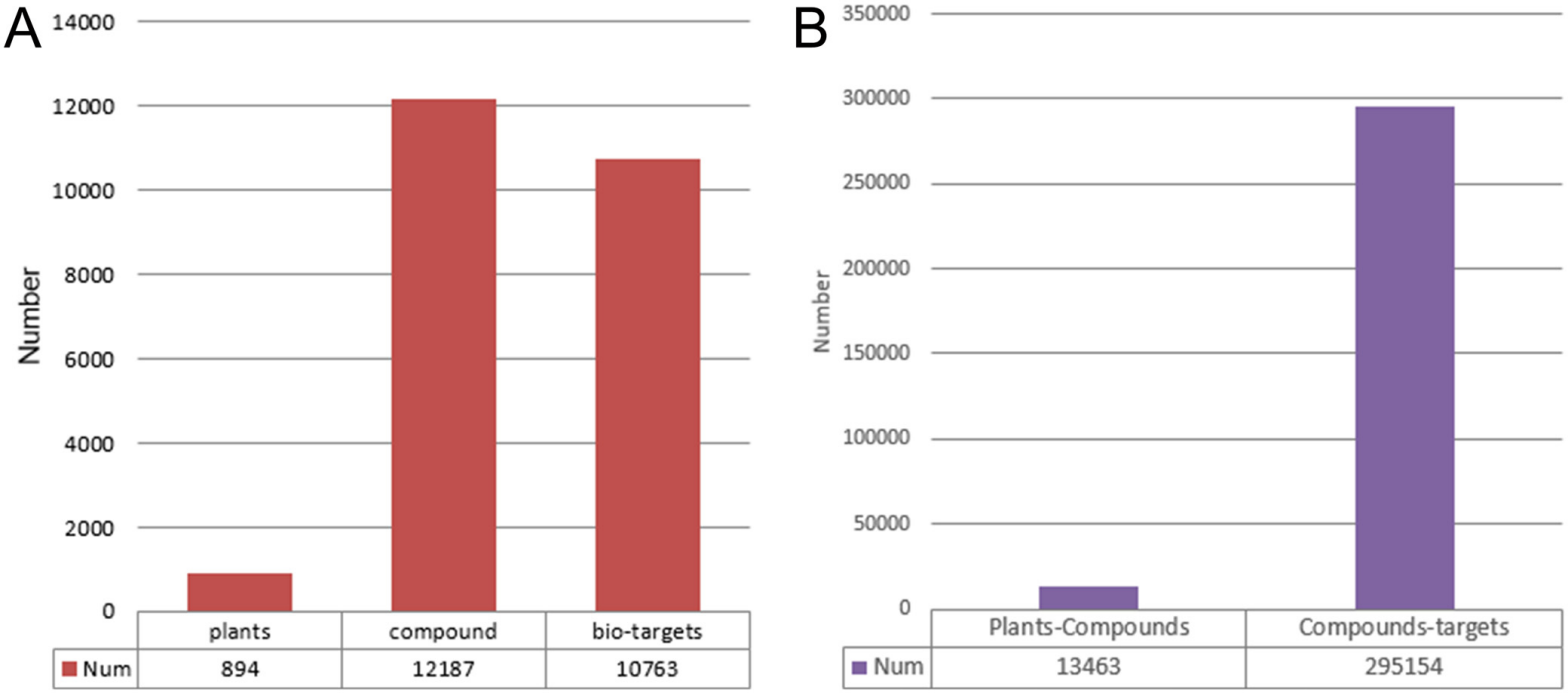
Summary of the data collections. (a) Data record numbers for Plants, Compounds and Bio-targets. (b) Data record reference quantities for Plants-Compounds and Compounds-Targets.

**Fig 2:**
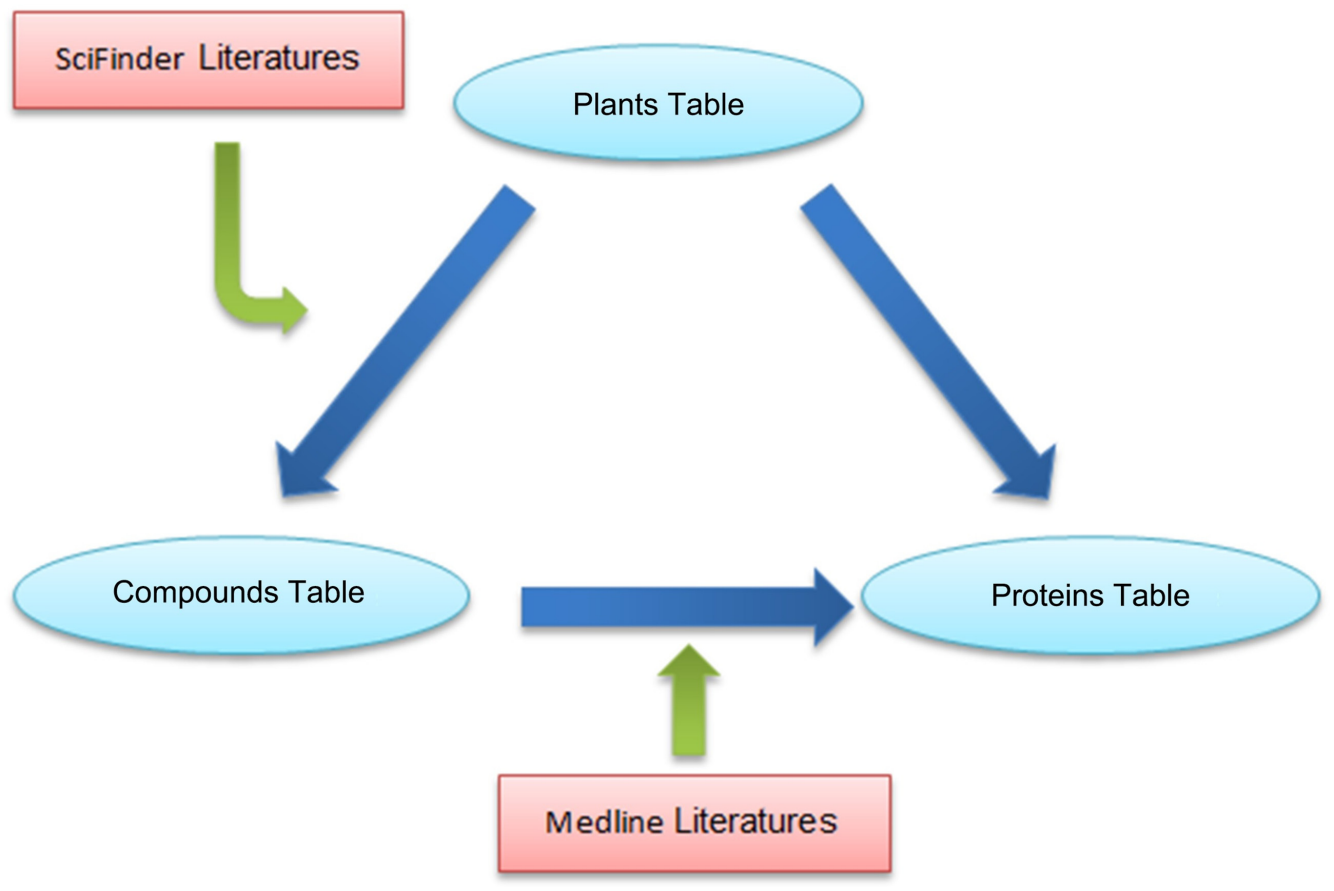
The relationship between data tables and data sources. Reference data for Plants-Compounds were collected from SciFinder Scholar, and reference data for Compounds-Targets were collected from PubMed MEDLINE. For medicinal plant researchers or traditional medicine developers, the relationship between plants and targets can be obtained from our database.

We gathered information on 894 medicinal plants from Chinese, Japanese, European, and American Pharmacopoeia (Fig A in S1 File). Text mining technologies were used to collect information on plant–compound inclusion relationships and compound–protein interactions from SciFinder Scholar and PubMed abstracts (Figs B and C in S1 File). We downloaded all MEDLINE abstracts listed on PubMed from 1970 to Nov 2015, as well as abstracts listed on SciFinder Scholar. After reviewing the plant–compound literature, we found that the titles of almost all the natural product chemistry studies have the similar style of expression, such as "Study on the chemical constituents of the herb *Mentha haplocalyx*". Thus, we retrieved studies using Latin name search terms: varieties and subspecies names + “chemical compositions,” “chemical constituents,” “chemical components,” and “chemical ingredients.” For each medical plant, we conducted separate searches using these four search terms. Finally, we obtained 13,463 non-redundant records, including information on paper titles, abstracts, authors, and publication dates. We constructed a dictionary of protein names that includes 25,995 protein records, and each protein has an average of nine aliases. We read a number of corpora (including GENETAG [19], BioInfer [20], and PennBioIE [21]) to create a compound–protein relation verb vocabulary list of 914 words [22]. We implemented the support vector machine (SVM) algorithm for text data mining using the Python programming language and the NLTK package. Every literature record extracted through text mining was manually curated. Thus, for 295,154 publications, 12,187 natural products and 10,763 potential bio-targets were identified (Fig 1). The relationships between natural products and potential bio-targets could be direct regulation or indirect effects. A node in the network contains detailed information (Entrez Gene ID, Gene Symbol, chromosome locations, Ensembl ID, OMIM ID, RefSeq Identifier, HGNC ID, UniProt ID), and almost every edge contains literature-based or scientific database-related evidence. Clicking on the edge provides more literature and supporting details. In addition, gene pathways, Gene Ontology (GO) data, and bio-network information are integrated in accordance with relevant bio-targets. TarNet currently includes rich information regarding plant–compound and compound–protein relationships. All data were stored in a SQLite file, which is a cross-platform lightweight database system without a separate server process that is easy to copy, move, and share. Fig 2 presents the relationships between the data tables and data sources.

To conduct a comprehensive and unbiased study of disease-PPI networks, we downloaded all human PPI data from seven major public PPI databases (BioGRID, BIND, DIP, HPRD, iRefWeb, IntAct, and MINT). First, we removed entries with “None” listed in the Entrez Gene ID column, and all of the data were then merged after removing the redundant data. To ensure high-quality results, we removed those records that only appeared in one of the above-mentioned database. To date, TarNet integrates 216,587 experimentally confirmed PPI data entries on 16,452 human proteins. A broad landscape of data on PPI was collected from major public databases (Table 1).

**Table 1:** A broad collection of data on PPI datasets.

Database	Raw Interactions	Proteins
BIND	13,034	3,082
BioGRID	221,260	15,341
DIP	4,150	2,443
HPRD	39,240	9,663
IntAct	101,129	11,197
iRefWeb	406,622	14,808
MINT-binary	31,252	6,181
MINT-complexes	3,479	1,727
MINT	39,125	8,120
All-Non-Redundant interaction Records	216,587	16452

Type 2 Diabetes mellitus (T2DM)-related protein data were collected from T2D-Db V2 databases [23], T2D@ZJU and OMIM. The most reliable datasets drawn from the literature were manually curated, extracted, and integrated into our web server to serve as an example. A total of 328 nodes and 1,229 edges were constructed in the T2D-Network.

### Processing method

Python was used as our main data processing programming language. We used the NetworkX (http://networkx.github.io/) python package for server computations and analyses. NetworkX provided algorithms and routines to create, manipulate, and examine the structures, dynamics, and functions of complex networks. Standard HTML4.01/CSS, Javascript and the CytoscapeWeb library [24] were used to browse and interact with networks. Users only require access to a standard web browser updated to the latest version.

## Results

**Fig 3:**
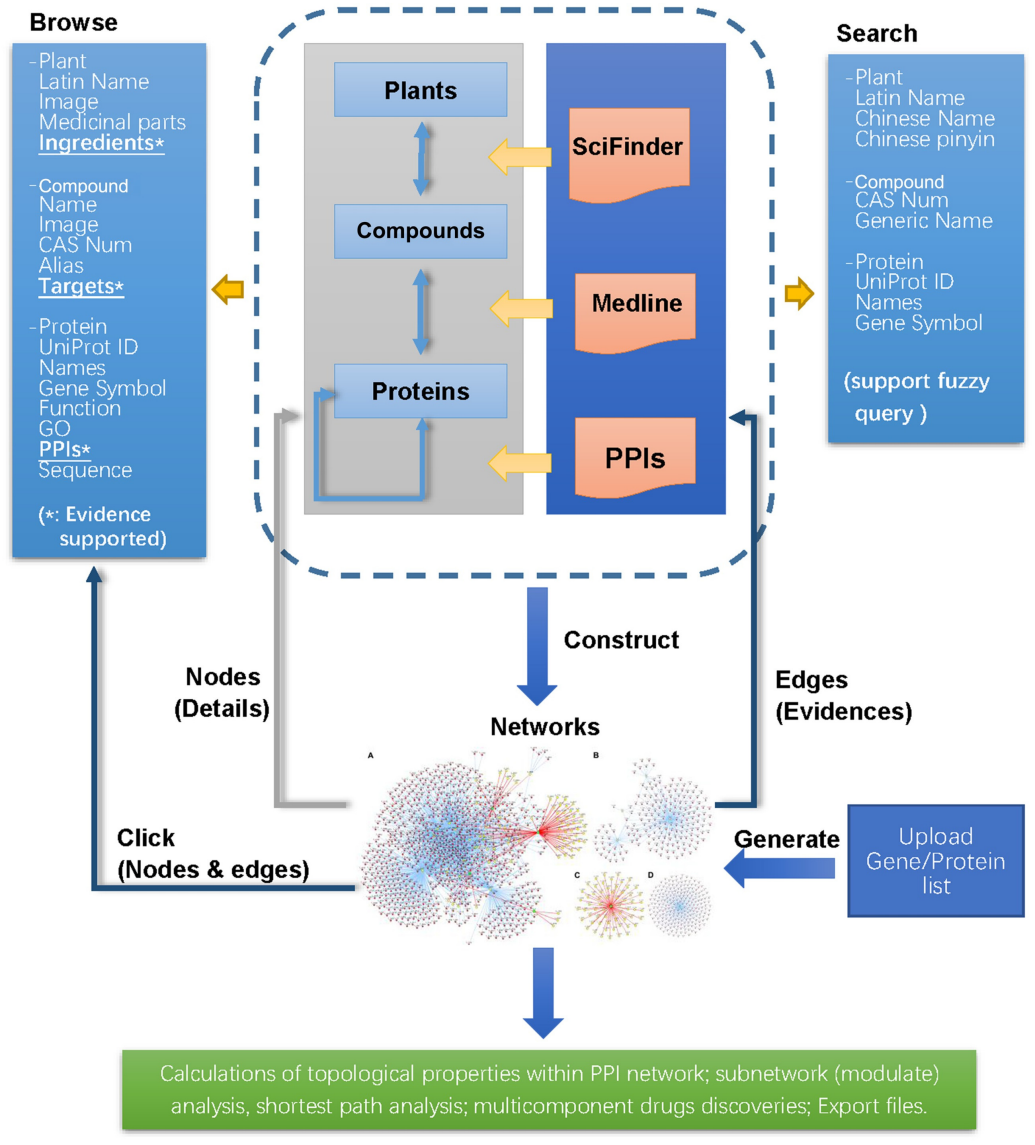
Graphical summary of major functions of the platform.

TarNet is freely available to all users and serves as a user-friendly web interface for users to browse or search for data and to interact with networks. Major functions of the platform are illustrated in Fig 3.

### Databases

**Fig 4:**
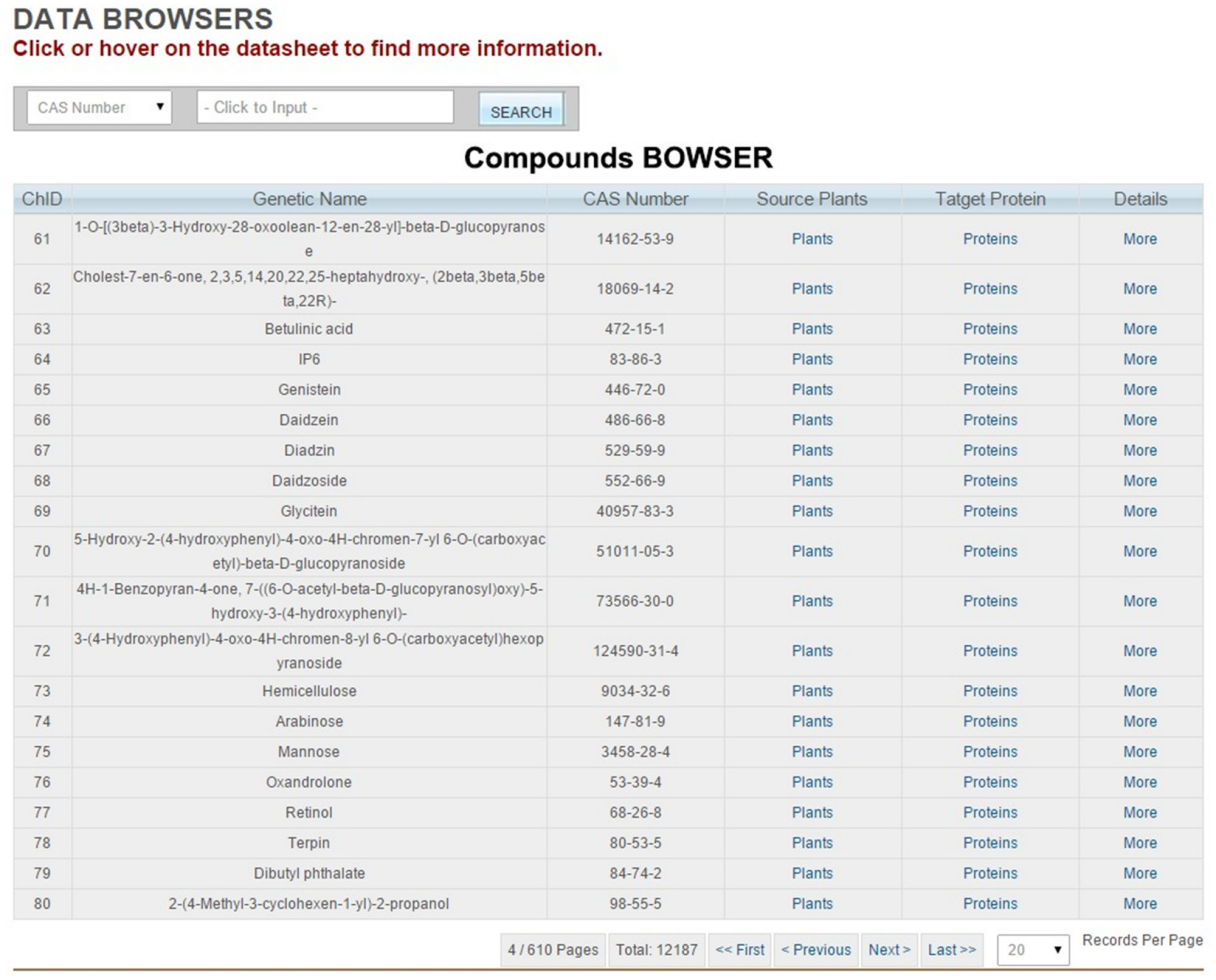
The interference of data tables (Compound Table used as an example).

Three database forms are available through TarNet menu bar: Plant, Compound, and Protein. Users can obtain information on their queries by searching through the database. All the database forms provide a search field with several optional search terms, and every record can be opened to access further information (Fig 4). On Plant Detail pages, users can view Latin names, images (plant pictures), the names of official medicinal parts, and ingredients. On Protein Detail pages, detailed protein information is provided (e.g., UniProt IDs, names, functions, pathways, GO data, and sequences). Compound data include names, CAS numbers, aliases, molecular details, and information on certain physical and chemical properties.

**Fig 5:**
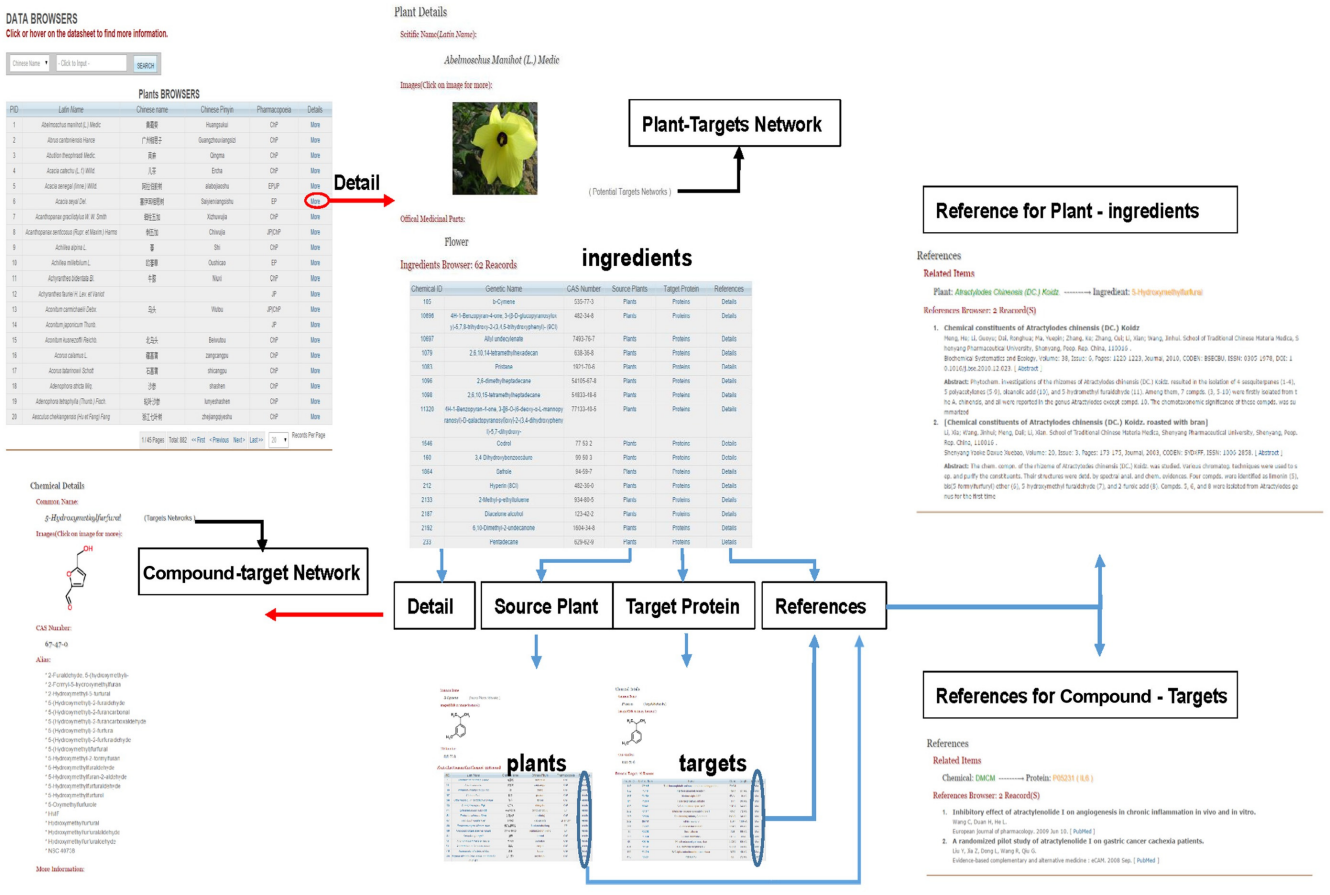
Data browsers for Plants and Compounds and links between different functions. Information on both plant–compound inclusion relationships and compound–target interactions is supported by study findings.

**Fig 6:**
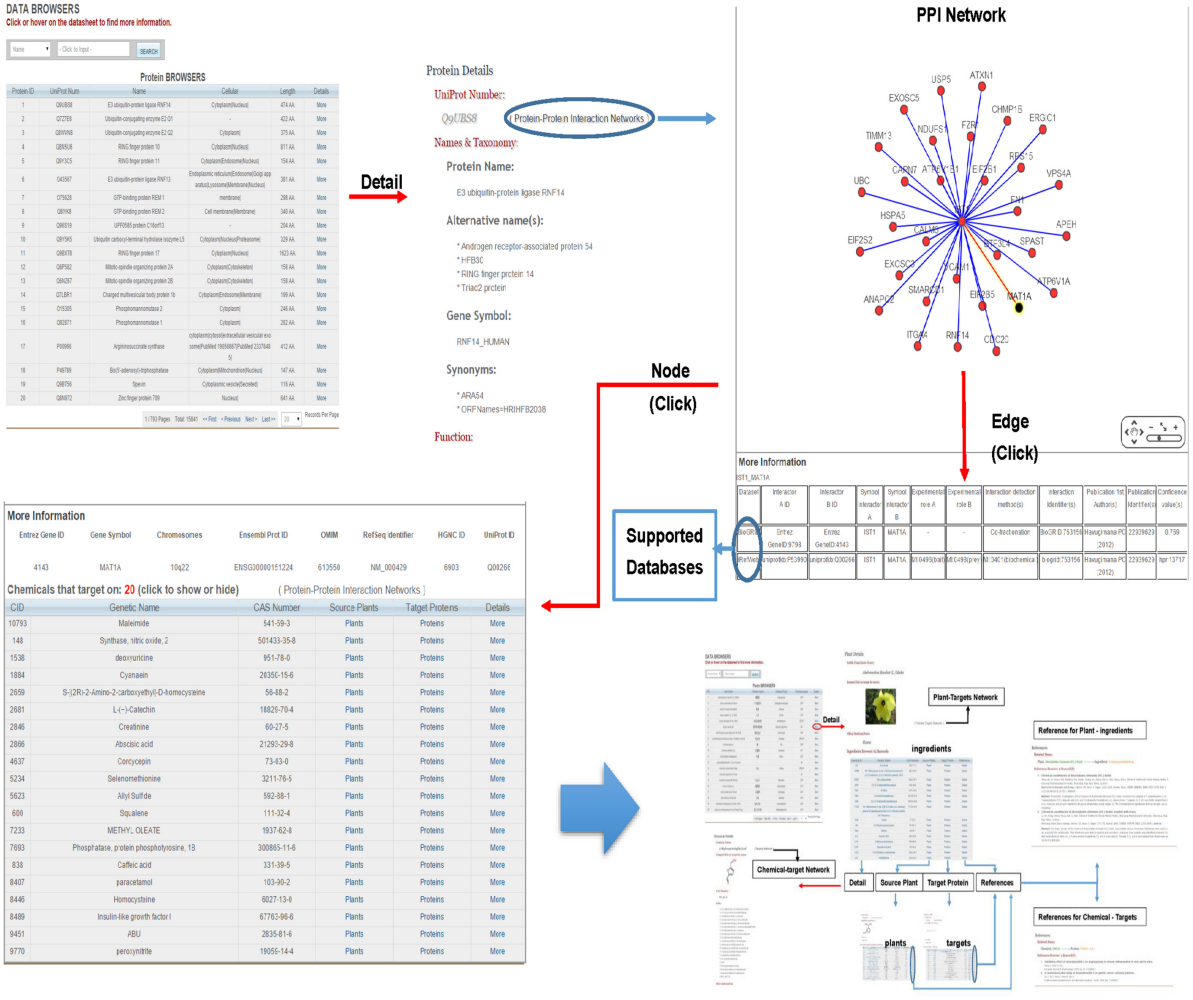
Data searches and browsers for Proteins and the construction of the PPI network. Nodes and edges can be clicked on to access more information on the network. Each edge is supported by public database records.

On each Plant Detail page, compounds are listed along the bottom in a table, and a supporting link to references is included for each record within each table for the identification of related publications in addition to targets listed on Compound Detail pages (Fig 5). On Protein Detail pages, a link is positioned beside the UniProt ID, which was used to build a PPI network associated with each protein. Nodes and edges are interactive, and more information is displayed below the graph by clicking on them (Fig 6).

**Fig 7:**
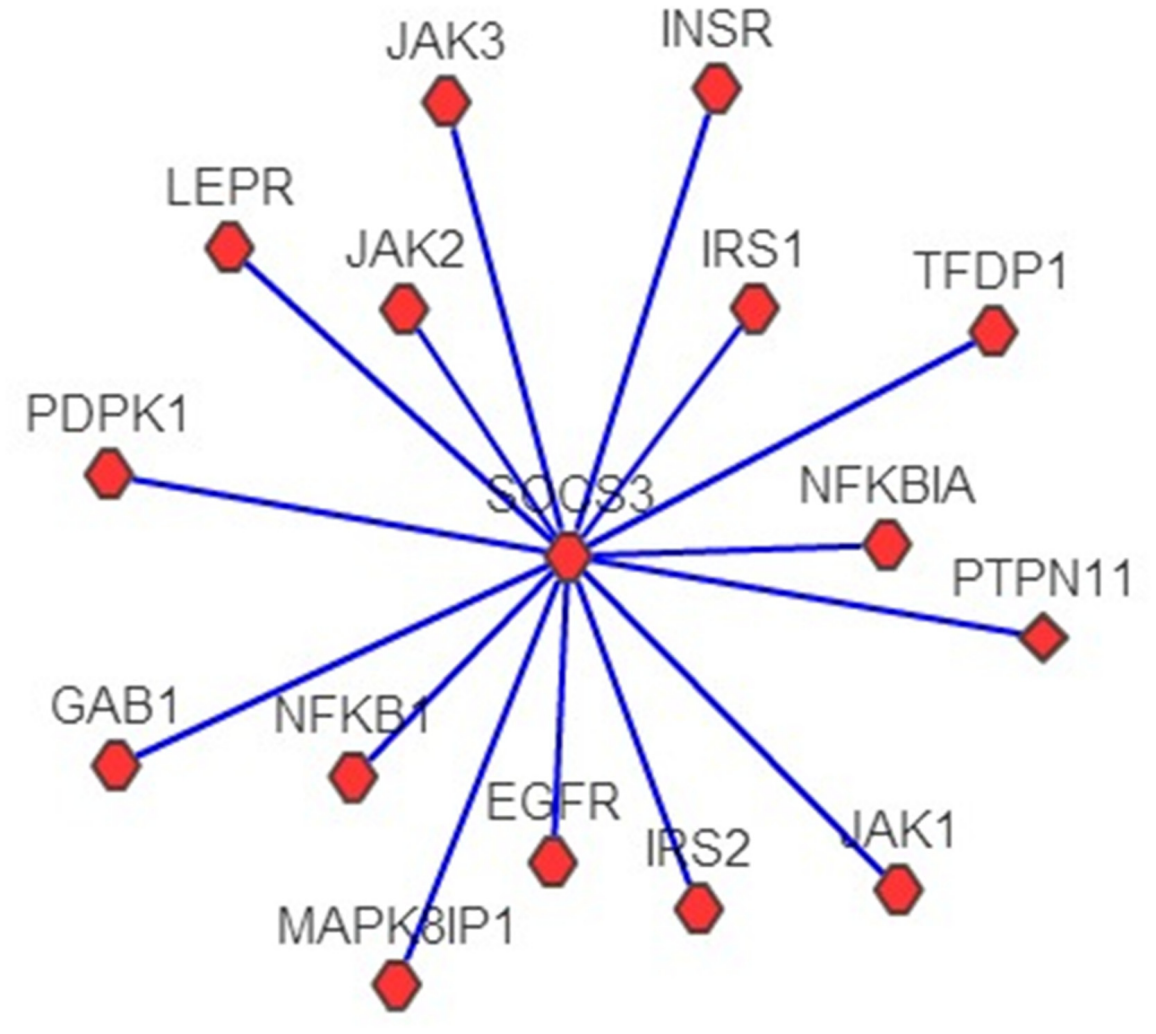
The construction of protein-protein interaction networks using ‘SOCS3.’

To demonstrate the utility of the search and network-construction functions and the associated knowledge discoveries, a case study was conducted. A search with ‘UniProtID: O14543’ used as a query keyword and with T2D-associated proteins used as the dataset resulted in the generation of a PPI network with 16 nodes (Fig 7) directly related to SOCS3. In cases of obesity, SOCS3 is upregulated in skeletal muscles, the liver, in adipose tissues and in the hypothalamus [25–28], and this upregulation is associated with increased inflammation. SOCS3 is a major negative regulator of insulin and leptin signaling [25] and is thought to contribute to the pathogenesis of insulin resistance. In adipocytes, SOCS3 deficiency increases insulin-stimulated IRS1 and IRS2 phosphorylation, whereas the overexpression of SOCS3 reduces both IRS1 protein levels and the phosphorylation of IRS1 and IRS2 [29]. The genetic deletion of SOCS3 in mouse liver results in increased insulin sensitivity levels due to increased levels of IRS1 phosphorylation [30]. SOCS3 co-immunoprecipitates with both INSR and IRS1 in skeletal muscles [31]. SOCS3 inhibits leptin signaling by binding to phosphorylated tyrosine residue in the leptin receptor [32]. SOCS3 is a major regulator of JAK signaling as it binds and inhibits catalytic domains of JAK2 via an evolutionarily conserved motif unique to JAKs [33]. Therapies aimed at inhibiting SOCS3 in skeletal muscle may help reverse glucose intolerance and insulin resistance [34]. NF-κB plays an important role in regulating PIM2 induced SOCS3 expression [35]. Therefore, NF-κB inhibition may serve as an alternative method to reduce SOCS3 expression. Moreover, salicylates inhibit NF-κB by inhibiting Iκ-B kinase-β [36–38]. A decline in SOCS3 expression may be involved in mechanisms of the anti-inflammatory drug through which it functions.

### PPI network construction

From the “Upload Center” menu, users may select a dataset ID and “Browse” a text file to upload a protein/gene list to “Generate” a network.

- File Type: The file uploaded must be a text file with a “.txt” extension.
- Content and Format: Protein or Gene lists should be submitted as a single column with no blank entries.

The graphical, interactive network associated with the uploaded protein/gene list is shown in the center of the screen. The user can then interact with the network (using the same method as that for Disease PPIs). In TarNet, SIF (TXT), PNG or XML (XGMML) file are available for download.

**Fig 8:**
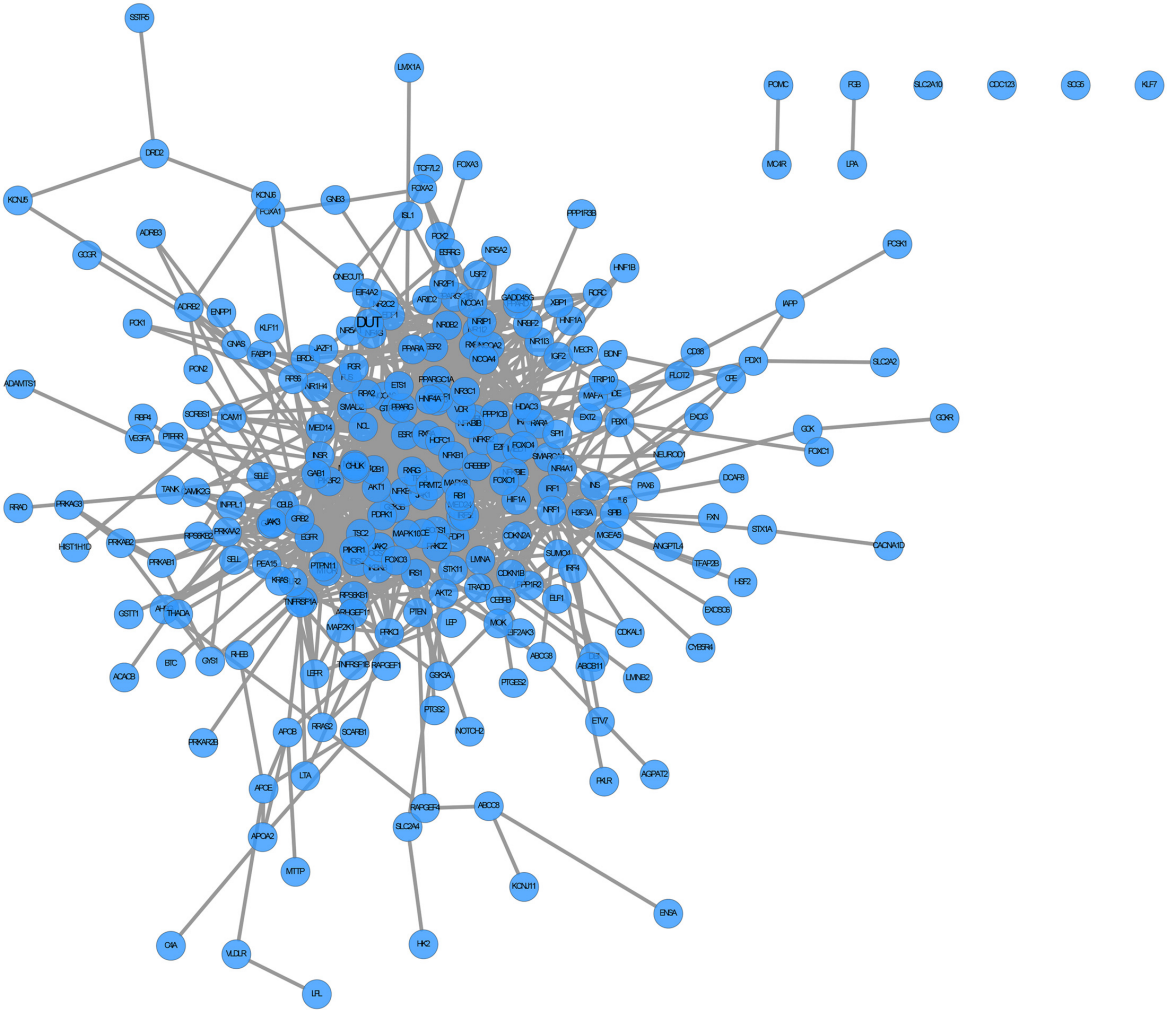
The protein-protein interaction network of T2D.

**Fig 9:**
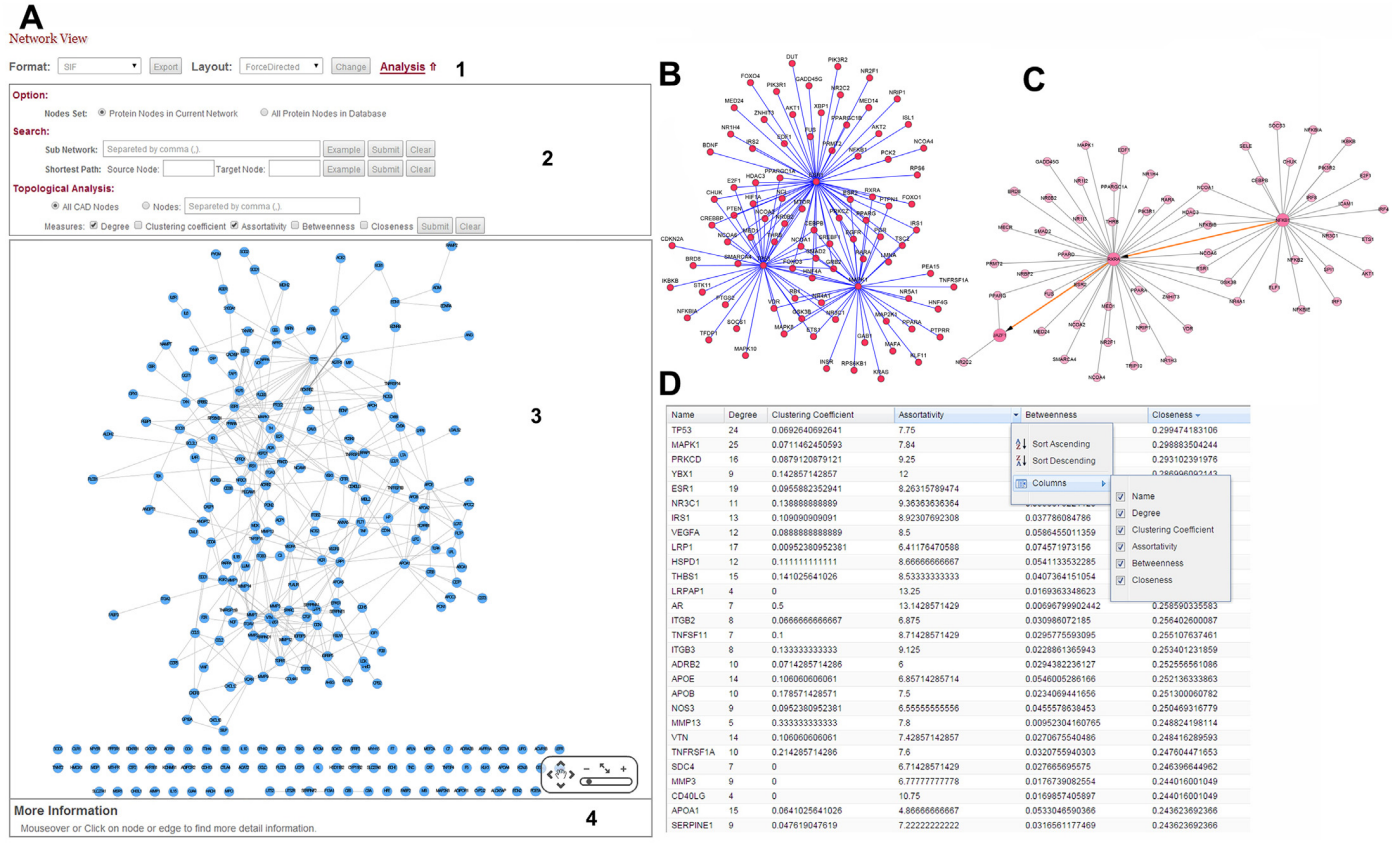
Interface and sample outputs of PPI networks. (A) PPI Network user interface: (1) Export and layout settings. (2) tool box, (3) network graph panel, and (4) information box. (B) Subnetwork graph constructed from randomly selected nodes in the TarNet database. (C) A sample shortest path found using TarNet from NFKB1 to JAZF1. (D) Results generated using the TarNet topological analysis tool are sorted in descending order according to the Closeness value of each node.

We integrated T2DM [23, 39] in TarNet (Fig 8). Users can conduct further analyses such as subnetwork analyses, shortest path analyses, and topological calculations to evaluate the significance of a specified node and PPI Networks that users have uploaded. Placing the computer mouse on a node causes the node to glow and its label to expand. Placing the computer mouse over an edge highlights the edge. Users can click on nodes and edges within a network to obtain detailed information on these features presented in an information box positioned at the bottom of the page. All nodes can be dragged by clicking and moving the mouse. In TarNet, five network measures (Degree, Clustering coefficient, Assortativity, Betweenness, and Closeness) are computed to evaluate the significance of a specified node according to a reference [40]. The measured results are presented in a table, and entries can be sorted, hidden or shown by clicking on various columns. The interface and sample outputs of PPI networks are presented in Fig 9.

We determined the topological properties of the T2DM network and sorted using the Betweenness measure. Betweenness is an indicator of a node's degree of centrality in a network and is equal to the number of shortest paths from all vertices to all others that pass through a node. A node with high betweenness has a considerable influence on the movement of items through a network based on the assumption that item transference follows the shortest paths. The top ranked genes were IRS1, TCF7L2, PPARA, and ENPP1, complementing previous findings. TCF7L2 is an important gene in determining T2DM susceptibility levels. Common variants of the TCF7L2 gene are strongly associated with risks of developing T2DM in multiethnic cohorts [41–44]. IRS1 is an intracellular substrate of the tyrosine kinase insulin receptor that participates in the insulin signal transduction pathway. Two mutations of the IRS1 gene (Gly972Arg and Ala513Pro) are related to delayed-type T2DM [45]. The ENPP1 gene was significantly correlated with insulin resistance in 1999 [46]. PPARA is a susceptibility gene for obesity, T2DM and insulin resistance [47, 48].

### Network pharmacology analysis

TarNet is designed to generate an analysis pipeline as a plant–compound–target (protein)–PPI network/disease or as a sub of this pipeline. In the process of network construction, compound-related proteins were first retrieved from the database. Then, these proteins were mapped to the PPI relationships datasets to obtain the interactive relations between them. Finally, compounds associated PPI networks were built. Edges that connect compounds and proteins and an inclusive relationship between plants and compounds were proven based on the literature. The connection between compounds and proteins may represent a relationship of direct regulation or indirect effects. TarNet can serve as a resource for systems biology and network pharmacology researchers [49–51]. This system will facilitate studies on drugs, and especially studies on herbal medicines or Traditional Chinese Medicines conducted at the system level [52–54].

**Fig 10:**
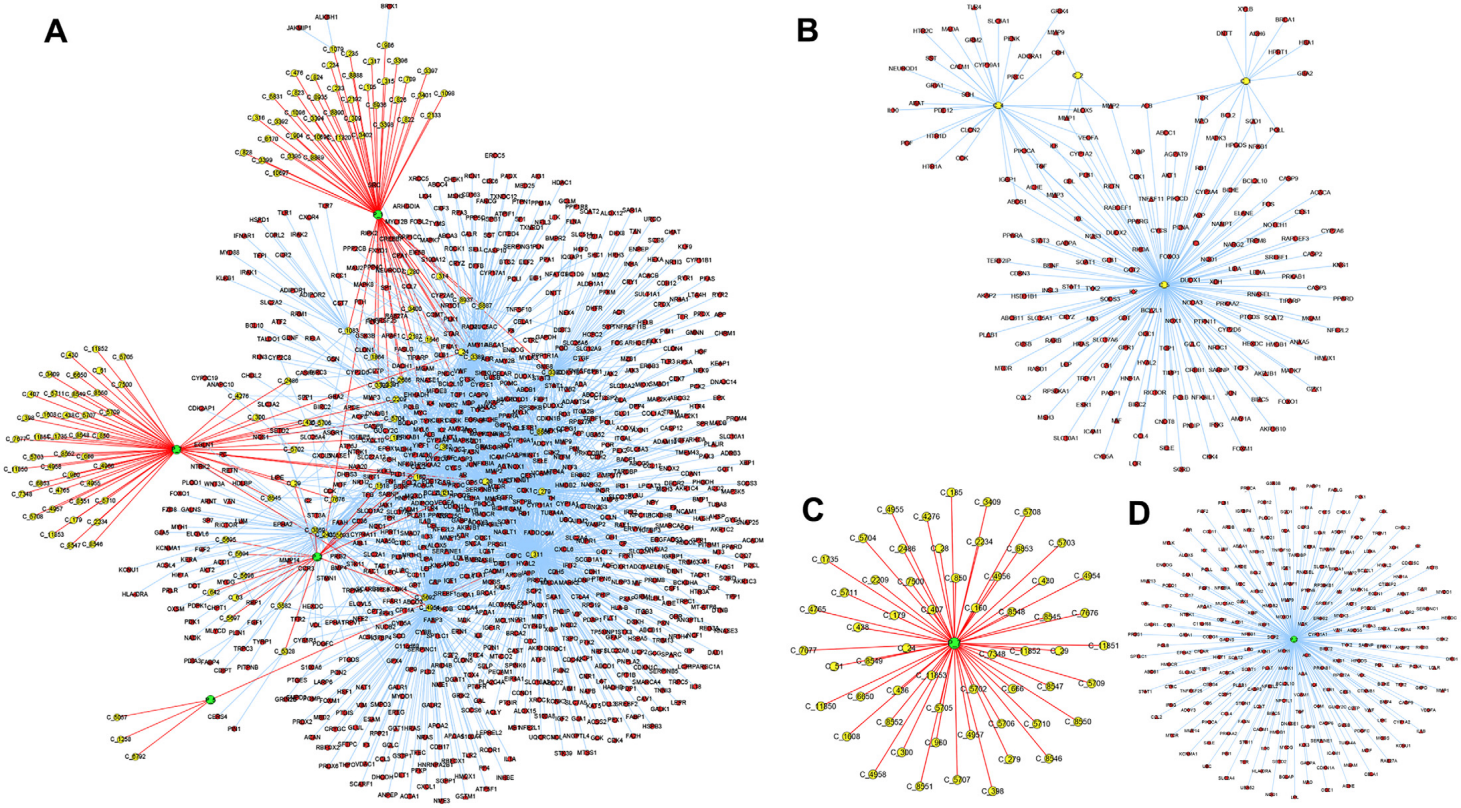
Sample outputs of Plant-Compound-Target networks. (COLOR: Green Nodes-Plants, Yellow Nodes-Compounds, Red Nodes-Target Proteins; SHAPE: Diamond-T1D, Hexagon-T2D, Triangle-CAD). (A) Plant-Compound-Target network selected from the TarNet database. (B) Compound-Target Network. (C) Plant and its ingredients. (D) Medicinal plant and its target.

Researchers are merely required to upload their protein or gene lists. PPI networks are calculated on the server end and are then sent back to the screen. Topological degree, clustering coefficient, associativity, betweenness, and node closeness properties in uploaded and entire human PPI networks can be determined, thus helping researchers to identify key nodes [50, 55]. Functions for obtaining subnetworks of one or more nodes and the shortest paths between nodes are available. Furthermore, researchers can determine whether certain compounds interact with uploaded proteins and how they engage with each other, thus helping researchers to identify potential drug targets for a natural product or herbal plant. For research on herbal medicine or Traditional Chinese Medicine, prescriptions given to patients often consist of several herbs that contain numerous compounds. TarNet can help researchers find potential targets of certain herbs or compound groups while generating illustrations of how prescriptions or multi-component drugs function, thus facilitating network pharmacology analyses and drug discovery [56, 57]. For drug and pharmacological researchers, TarNet will be considered one of the most helpful tools available. For example, berberine was supposedly used in ancient China as a folk medicine that was found in such plants such as *Berberis*. By searching TarNet, 434 records of potential targets of berberine were obtained, and GO analysis was conducted on these proteins. The result showed that these related proteins were associated with the cell cycle and apoptotic process, steroid metabolic process, carbohydrate metabolic process, fatty acid metabolic process, and lipid metabolic processes, etc. Notoginseng triterpenes are the main components of Radix Notoginseng including Ginsenoside Rg1, Ginsenoside Rb1 and Notoginsenoside R1. We investigated whether notoginseng triterpenes could be a solution for ischemic stroke. Through the 'Network Pharmacology' analysis, TarNet can build a shared-network of all related proteins, and all the relationships are supported by published studies. Ischemic-stroke-related proteins were retrieved from OMIM, and mapped to the shared-network of notoginseng triterpenes. Notoginseng triterpenes share many proteins that are related to ischemic stroke. Utilizing TarNet, researchers can discover new uses for old drugs, conduct drug toxicology and side effect studies, and develop innovative drugs (multi-ingredient products or drug groups). The sample outputs of Plant–Compound–Target networks are presented in Fig 10.

### Features

1. Extensive information on plants, compounds, proteins (nodes in networks) and references is provided.
2. Almost every record on plant–compound and compound–protein relationships and PPI (network edges) is supported by literature-based or scientific database-derived evidence.
3. Users can upload Protein/Gene lists to build PPI networks for analyses with different ID types: official gene symbols, Entrez Gene IDs, UniProt IDs, RefSeq IDs, Ensembl IDs, and HGNC IDs.
4. Nodes and edges are interactive and thus provide additional detailed information.
5. Calculations of topological properties within diseased-PPI networks or entire human PPIs, subnetwork (modulate) analyses, and shortest path analyses.
6. Customization using different layouts, zooming options, and highlight network visualization features.
7. Network editing (i.e., node deletion, first neighbor highlighting, and edge deletion).
8. Export networks as PNG (Open with a web browser), SIF (TXT), and XML (XGMML) files.
9. Substructure search is supported for compound searches.

A comparison with other similar tools has been conducted, and the detailed information is listed in S1 Table. Although these tools are very good databases, there are still some shortcomings. TCMSP (lsp.nwsuaf.edu.cn/tcmsp.php) is a database that includes chemicals, targets and drug–target networks. Data is integrated from other databases. Users can retrieve data by typing an herb name, chemical name or a target name. TCMID (www.megabionet.org/tcmid/) provides information on six different areas (formulae, herb, compound, disease, drug and target) and the links between them. All the information is listed as a text or table. ASDB (www.rcdd.org.cn/asdb) is an open scaffold-orientated database through systematic annotations. BATMANTCM (bionet.ncpsb.org/batman-tcm/) is an online bioinformatics analysis tool specially designed for the investigation of the molecular mechanisms of TCMs, mainly based on target predictions for the ingredient of TCMs. All the above tools provide no information on PPI networks. None of the tools provides functions for uploading disease-associated proteins and disease-associated PPI network construction. Only a few of them provided topological analysis. TCMSP only allows users to query a single herb, a query for formulae or multiple herbs is not supported. ASDB provides no information on herbs or medicinal plants. BATMANTCM focuses on the molecular mechanisms of existing TCM formulae, and does not provide customized selection of herbs, which may limit discoveries of new formulae or new herb combinations. There are some deficiencies in our TarNet database; for example, disease and formula queries are not currently supported. In our future work, we will address these deficiencies.

## Discussion

The material foundations and mechanisms of herbal medicine are essential to the standardization of drugs quality levels, the discovery of new drugs, and the modernization of traditional medicine. Although research on herbal medicine material foundations has been conducted for approximately 100 years, the material foundations of most herbal medicines remain unclear. The effective components of herbal medicine are complex, with various ingredients that may act on multiple targets. Effective techniques that allow researchers to acquire more insight into these components are lacking. The current expansion of systems biology and network pharmacology has resulted in numerous opportunities for research on herbal medicines and complex diseases [4, 52].

Nodes for which knowledge remains limited have fewer connections with other entities. However, some nodes are proposed to be associated with enhanced disease-related risks. Connections with PPI datasets provide valuable clues, allowing researchers to propose hypotheses that can be tested through further experimentation.

As complex diseases often result from network changes that disturb the system balance, effective drugs should weaken these changes and recover the balance. Network pharmacology techniques have been proposed based on theories of network biology and biological system balance to provide new ideas and research strategies for novel drug research and development. “Magic bullets” will be replaced by “shotguns” [4]. Consistent with holistic and dynamic characteristics, the field of network pharmacology advocates for the use of multiple targets for multiple methods of medication. Over the course of traditional medicine modernization, a number of researchers have successfully explored the foundations of drugs, revealing comprehensive effects generated from multi-components, multi-targets, and multiple uses of herbal medicines.

## Conclusion

To assist network pharmacology and systems biology researchers, we built TarNet, a powerful data search tool that offers extensive data resources on plant-compound-target relationships based on evidence and an analytical and visual exploration platform for diseases associated with PPI networks. Using TarNet, researchers can obtain information on medicinal plants and compounds as well as biological information on specific genes or proteins from a network perspective (e.g., the relationship between a precise gene and other genes and the importance of a specific node to the network). Operational tools can be accessed by users to input their own protein or gene datasets and thereby construct and analyze the network. This intuitive user interface and extensive network information database on proteins and genes make TarNet a useful tool that can help researchers generate and investigate hypotheses. TarNet is freely available and completely accessible to all users.

## Availability and Requirements

TarNet is free and available to all users without login requirements at http://www.herbbol.org:8001/tarnet. Users must have access to a modern browser with the Flash plugin installed. Cytoscape Web is tested on the latest version of Chrome.

## Data Access

The minimal data set underlying the findings have been uploaded to the TarNet website at http://www.herbbol.org:8001/tarnet/data/Data.db. We also deposit our data into Figshare (https://figshare.com/s/7b5e1d93f2b6cca89e8e).

## Supporting Information

S1 File Flowcharts of data collection of medicinal plants, compounds, proteins. (PDF)

S1 Table The comparison between TarNet and other similar databases. (PDF)

## References

[1] Yıldırım MA, Goh K-I, Cusick ME, Barabási A-L, Vidal M. Drug—target network. Nat Biotechnol. 2007;25(10):1119–26. 17921997 10.1038/nbt1338

[2] Overington JP, Al-Lazikani B, Hopkins AL. How many drug targets are there? Nat Rev Drug Discov. 2006;5(12):993–6. 17139284 10.1038/nrd2199

[3] Zheng M, Liu X, Xu Y, Li H, Luo C, Jiang H. Computational methods for drug design and discovery: focus on China. Trends Pharmacol Sci. 2013;34(10):549–59. 10.1016/j.tips.2013.08.004 24035675 PMC7126378

[4] Hopkins AL. Network pharmacology: the next paradigm in drug discovery. Nat Chem Biol. 2008;4(11):682–90. 10.1038/nchembio.118 18936753

[5] Drews J. Drug discovery: a historical perspective. Science. 2000;287(5460):1960–4. 10720314 10.1126/science.287.5460.1960

[6] Costello JC, Heiser LM, Georgii E, Gonen M, Menden MP, Wang NJ, et al A community effort to assess and improve drug sensitivity prediction algorithms. 2014;32(12):1202–12. 10.1038/nbt.2877 . 24880487 PMC4547623

[7] Keiser MJ, Setola V, Irwin JJ, Laggner C, Abbas AI, Hufeisen SJ, et al Predicting new molecular targets for known drugs. Nature. 2009;462(7270):175–81. 10.1038/nature08506 19881490 PMC2784146

[8] Zheng M, Liu X, Xu Y, Li H, Luo C, Jiang H. Computational methods for drug design and discovery: focus on China. Trends in pharmacological sciences. 2013;34(10):549–59. 10.1016/j.tips.2013.08.004 24035675 PMC7126378

[9] Shao L, Zhang B. Traditional Chinese medicine network pharmacology: theory, methodology and application. Chin J Nat Med. 2013;11(2):110–20. 10.1016/S1875-5364(13)60037-0 23787177

[10] Tao W, Xu X, Wang X, Li B, Wang Y, Li Y, et al Network pharmacology-based prediction of the active ingredients and potential targets of Chinese herbal Radix Curcumae formula for application to cardiovascular disease. J Ethnopharmacol. 2013;145(1):1–10. 10.1016/j.jep.2012.09.051 23142198

[11] Han J-D, Bertin N, Hao T, Goldberg DS, Berriz GF, Zhang LV, et al Evidence for dynamically organized modularity in the yeast protein–protein interaction network. Nature. 2004;430(6995):88–93. 15190252 10.1038/nature02555

[12] Xia J, Benner MJ, Hancock RE. NetworkAnalyst—integrative approaches for protein-protein interaction network analysis and visual exploration. Nucleic Acids Res. 2014;42(Web Server issue):W167–74. Epub 2014/05/28. 10.1093/nar/gku443 ; PubMed Central PMCID: PMCPmc4086107. 24861621 PMC4086107

[13] Arrell D, Terzic A. Network systems biology for drug discovery. Clin Pharmacol Ther. 2010;88(1):120–5. 10.1038/clpt.2010.91 20520604

[14] Ideker T, Sharan R. Protein networks in disease. Genome research. 2008;18(4):644–52. Epub 2008/04/03. 10.1101/gr.071852.107 ; PubMed Central PMCID: PMCPmc3863981. 18381899 PMC3863981

[15] Jiang X, Gold D, Kolaczyk ED. Network-based auto-probit modeling for protein function prediction. Biometrics. 2011;67(3):958–66. Epub 2010/12/08. 10.1111/j.1541-0420.2010.01519.x ; PubMed Central PMCID: PMCPmc3116961. 21133881 PMC3116961

[16] Liu H, Liu W, Liao Y, Cheng L, Liu Q, Ren X, et al CADgene: a comprehensive database for coronary artery disease genes. Nucleic Acids Res. 2011;39(Database issue):D991–6. Epub 2010/11/04. 10.1093/nar/gkq1106 ; PubMed Central PMCID: PMCPmc3013698. 21045063 PMC3013698

[17] Xiong J, Liu J, Rayner S, Li Y, Chen S. Protein-protein interaction reveals synergistic discrimination of cancer phenotype. Cancer informatics. 2010;9:61–6. Epub 2010/05/12. ; PubMed Central PMCID: PMCPmc2865773. 20458363 10.4137/cin.s3899 PMC2865773

[18] Yang D, Xie P, Liu Z. Ischemia/reperfusion-induced MKP-3 impairs endothelial NO formation via inactivation of ERK1/2 pathway. PloS one. 2012;7(7):e42076 Epub 2012/08/01. 10.1371/journal.pone.0042076 ; PubMed Central PMCID: PMCPmc3407110. 22848708 PMC3407110

[19] Tanabe L, Xie N, Thom LH, Matten W, Wilbur WJ. GENETAG: a tagged corpus for gene/protein named entity recognition. BMC bioinformatics. 2005;6(Suppl 1):S3 15960837 10.1186/1471-2105-6-S1-S3 PMC1869017

[20] Pyysalo S, Ginter F, Heimonen J, Björne J, Boberg J, Järvinen J, et al BioInfer: a corpus for information extraction in the biomedical domain. BMC bioinformatics. 2007;8(1):50. 17291334 10.1186/1471-2105-8-50 PMC1808065

[21] Liberman M, Mandel M. PennBioIE Oncology 1.0 Philadelphia: Linguistic Data Consortium; 2008 [cited 2013 Oct. 12]. Available from: https://catalog.ldc.upenn.edu/LDC2008T21.

[22] Zhang L, Berleant D, Ding J, Cao T, Syrkin Wurtele E. PathBinder–text empirics and automatic extraction of biomolecular interactions. BMC Bioinformatics. 2009;10(Suppl 11):S18–S. 10.1186/1471-2105-10-S11-S18 PMC3226189. 19811683 PMC3226189

[23] Agrawal S, Dimitrova N, Nathan P, Udayakumar K, Lakshmi SS, Sriram S, et al T2D-Db: an integrated platform to study the molecular basis of Type 2 diabetes. BMC Genomics. 2008;9:320 Epub 2008/07/09. 10.1186/1471-2164-9-320 ; PubMed Central PMCID: PMCPmc2491641. 18605991 PMC2491641

[24] Lopes CT, Franz M, Kazi F, Donaldson SL, Morris Q, Bader GD. Cytoscape Web: an interactive web-based network browser. Bioinformatics. 2010;26(18):2347–8. 10.1093/bioinformatics/btq430 20656902 PMC2935447

[25] Yang Z, Hulver M, McMillan RP, Cai L, Kershaw EE, Yu L, et al Regulation of insulin and leptin signaling by muscle suppressor of cytokine signaling 3 (SOCS3). PloS one. 2012;7(10):e47493 Epub 2012/11/02. 10.1371/journal.pone.0047493 ; PubMed Central PMCID: PMCPmc3480378. 23115649 PMC3480378

[26] Gu H, Liu L, Ma S, Liu Y, Ren Y, Zhai L, et al Inhibition of SOCS-3 in adipocytes of rats with diet-induced obesity increases leptin-mediated fatty acid oxidation. Endocrine. 2009;36(3):546–54. Epub 2009/10/29. 10.1007/s12020-009-9253-4 . 19862646

[27] Reed AS, Unger EK, Olofsson LE, Piper ML, Myers MG Jr., Xu AW. Functional role of suppressor of cytokine signaling 3 upregulation in hypothalamic leptin resistance and long-term energy homeostasis. Diabetes. 2010;59(4):894–906. 10.2337/db09-1024 20068134 PMC2844837

[28] Emanuelli B, Peraldi P, Filloux C, Chavey C, Freidinger K, Hilton DJ, et al SOCS-3 inhibits insulin signaling and is up-regulated in response to tumor necrosis factor-alpha in the adipose tissue of obese mice. The Journal of biological chemistry. 2001;276(51):47944–9. Epub 2001/10/18. 11604392 10.1074/jbc.M104602200

[29] Shi H, Cave B, Inouye K, Bjorbaek C, Flier JS. Overexpression of suppressor of cytokine signaling 3 in adipose tissue causes local but not systemic insulin resistance. Diabetes. 2006;55(3):699–707. . 16505233 10.2337/diabetes.55.03.06.db05-0841

[30] Sachithanandan N, Fam BC, Fynch S, Dzamko N, Watt MJ, Wormald S, et al Liver-specific suppressor of cytokine signaling-3 deletion in mice enhances hepatic insulin sensitivity and lipogenesis resulting in fatty liver and obesity. Hepatology. 2010;52(5):1632–42. 10.1002/hep.23861 . 20799351

[31] Yaspelkis BB 3rd, Kvasha IA, Figueroa TY. High-fat feeding increases insulin receptor and IRS-1 coimmunoprecipitation with SOCS-3, IKKalpha/beta phosphorylation and decreases PI-3 kinase activity in muscle. American journal of physiology Regulatory, integrative and comparative physiology. 2009;296(6):R1709–15. 10.1152/ajpregu.00117.2009 19386987 PMC2692786

[32] Bjorbak C, Lavery HJ, Bates SH, Olson RK, Davis SM, Flier JS, et al SOCS3 mediates feedback inhibition of the leptin receptor via Tyr985. The Journal of biological chemistry. 2000;275(51):40649–57. 10.1074/jbc.M007577200 . 11018044

[33] Babon JJ, Kershaw NJ, Murphy JM, Varghese LN, Laktyushin A, Young SN, et al Suppression of cytokine signaling by SOCS3: characterization of the mode of inhibition and the basis of its specificity. Immunity. 2012;36(2):239–50. 10.1016/j.immuni.2011.12.015 22342841 PMC3299805

[34] Jorgensen SB, O'Neill HM, Sylow L, Honeyman J, Hewitt KA, Palanivel R, et al Deletion of skeletal muscle SOCS3 prevents insulin resistance in obesity. Diabetes. 2013;62(1):56–64. 10.2337/db12-0443 22961088 PMC3526029

[35] Narayana Y, Bansal K, Sinha AY, Kapoor N, Puzo G, Gilleron M, et al SOCS3 expression induced by PIM2 requires PKC and PI3K signaling. Molecular immunology. 2009;46(15):2947–54. 10.1016/j.molimm.2009.06.019 . 19608279

[36] Hundal RS, Petersen KF, Mayerson AB, Randhawa PS, Inzucchi S, Shoelson SE, et al Mechanism by which high-dose aspirin improves glucose metabolism in type 2 diabetes. The Journal of clinical investigation. 2002;109(10):1321–6. Epub 2002/05/22. 10.1172/jci14955 ; PubMed Central PMCID: PMCPmc150979. 12021247 PMC150979

[37] Yuan M, Konstantopoulos N, Lee J, Hansen L, Li ZW, Karin M, et al Reversal of obesity- and diet-induced insulin resistance with salicylates or targeted disruption of Ikkbeta. Science. 2001;293(5535):1673–7. 10.1126/science.1061620 . 11533494

[38] Fleischman A, Shoelson SE, Bernier R, Goldfine AB. Salsalate improves glycemia and inflammatory parameters in obese young adults. Diabetes care. 2008;31(2):289–94. 10.2337/dc07-1338 17959861 PMC3226794

[39] Hulbert EM, Smink LJ, Adlem EC, Allen JE, Burdick DB, Burren OS, et al T1DBase: integration and presentation of complex data for type 1 diabetes research. Nucleic acids research. 2007;35(suppl 1):D742–D6. 17169983 10.1093/nar/gkl933 PMC1781218

[40] Yang Z, Yang J, Liu W, Wu L, Xing L, Wang Y, et al T2D@ ZJU: a knowledgebase integrating heterogeneous connections associated with type 2 diabetes mellitus. Database. 2013;2013:bat052 10.1093/database/bat052 23846596 PMC3708620

[41] Tong Y, Lin Y, Zhang Y, Yang J, Zhang Y, Liu H, et al Association between TCF7L2 gene polymorphisms and susceptibility to type 2 diabetes mellitus: a large Human Genome Epidemiology (HuGE) review and meta-analysis. BMC medical genetics. 2009;10:15 Epub 2009/02/21. 10.1186/1471-2350-10-15 ; PubMed Central PMCID: PMCPmc2653476. 19228405 PMC2653476

[42] Sladek R, Rocheleau G, Rung J, Dina C, Shen L, Serre D, et al A genome-wide association study identifies novel risk loci for type 2 diabetes. Nature. 2007;445(7130):881–5. 10.1038/nature05616 . 17293876

[43] Weedon MN, McCarthy MI, Hitman G, Walker M, Groves CJ, Zeggini E, et al Combining information from common type 2 diabetes risk polymorphisms improves disease prediction. PLoS medicine. 2006;3(10):e374 10.1371/journal.pmed.0030374 17020404 PMC1584415

[44] Loos RJ, Franks PW, Francis RW, Barroso I, Gribble FM, Savage DB, et al TCF7L2 polymorphisms modulate proinsulin levels and beta-cell function in a British Europid population. Diabetes. 2007;56(7):1943–7. 10.2337/db07-0055 17416797 PMC2668957

[45] Arikoglu H, Aksoy Hepdogru M, Erkoc Kaya D, Asik A, Ipekci SH, Iscioglu F. IRS1 gene polymorphisms Gly972Arg and Ala513Pro are not associated with insulin resistance and type 2 diabetes risk in non-obese Turkish population. Meta gene. 2014;2:579–85. Epub 2015/01/22. 10.1016/j.mgene.2014.07.008 ; PubMed Central PMCID: PMCPmc4287848. 25606440 PMC4287848

[46] Pizzuti A, Frittitta L, Argiolas A, Baratta R, Goldfine ID, Bozzali M, et al A polymorphism (K121Q) of the human glycoprotein PC-1 gene coding region is strongly associated with insulin resistance. Diabetes. 1999;48(9):1881–4. . 10480624 10.2337/diabetes.48.9.1881

[47] Wang Z, Wang Y-y. Modular pharmacology: deciphering the interacting structural organization of the targeted networks. Drug discovery today. 2013;18(11):560–6. 23353707 10.1016/j.drudis.2013.01.009

[48] Lopez-Alarcon M, Rodriguez-Cruz M, Vital-Reyes VS, Zavala-Ortega MI, Hinojosa-Cruz JC, Canizales-Quinteros S, et al PPARgamma2 Pro12Ala polymorphism is associated with improved lipoprotein lipase functioning in adipose tissue of insulin resistant obese women. Gene. 2012;511(2):404–10. Epub 2012/10/06. 10.1016/j.gene.2012.09.057 . 23036713

[49] Ren G, Liu Z. NetCAD: a network analysis tool for coronary artery disease-associated PPI network. Bioinformatics. 2013;29(2):279–80. 10.1093/bioinformatics/bts666 23162052

[50] Seebacher J, Gavin A-C. SnapShot: Protein-protein interaction networks. Cell. 2011;144(6):1000-. e1 10.1016/j.cell.2011.02.025 21414489

[51] Wang Z, Wang Y-y. Modular pharmacology: deciphering the interacting structural organization of the targeted networks. Drug Discov Today. 2013;18(11):560–6. 23353707 10.1016/j.drudis.2013.01.009

[52] Zhao S, Iyengar R. Systems pharmacology: network analysis to identify multiscale mechanisms of drug action. Annu Rev Pharmacol Toxicol. 2012;52:505–21. 10.1146/annurev-pharmtox-010611-134520 PMC3619403. 22235860 PMC3619403

[53] Yamanishi Y, Kotera M, Moriya Y, Sawada R, Kanehisa M, Goto S. DINIES: drug–target interaction network inference engine based on supervised analysis. Nucleic acids research. 2014;42(Web Server issue):W39–W45. 10.1093/nar/gku337 PMC4086078. 24838565 PMC4086078

[54] Zhao J, Jiang P, Zhang W. Molecular networks for the study of TCM pharmacology. Brief Bioinform. 2010;11(4):417–30. 10.1093/bib/bbp063 20038567

[55] Davis D, Yaveroglu ON, Malod-Dognin N, Stojmirovic A, Przulj N. Topology-function conservation in protein-protein interaction networks. Bioinformatics. 2015;31(10):1632–9. Epub 2015/01/23. 10.1093/bioinformatics/btv026 ; PubMed Central PMCID: PMCPmc4426845. 25609797 PMC4426845

[56] Yamada T, Bork P. Evolution of biomolecular networks—lessons from metabolic and protein interactions. Nat Rev Mol Cell Biol. 2009;10(11):791–803. 10.1038/nrm2787 19851337

[57] Barabási A-L, Gulbahce N, Loscalzo J. Network medicine: a network-based approach to human disease. Nat Rev Genet. 2011;12(1):56–68. 10.1038/nrg2918 21164525 PMC3140052

