# Supplementary material for: TarNet: An Evidence-Based Database for Natural Medicine Research
Source: PLoS One. 2016 Jun 23;11(6):e0157222. doi: 10.1371/journal.pone.0157222 (PMC4919029; doi:10.1371/journal.pone.0157222)
Supplement: S1 File — (PDF) [file pone.0157222.s001.pdf]

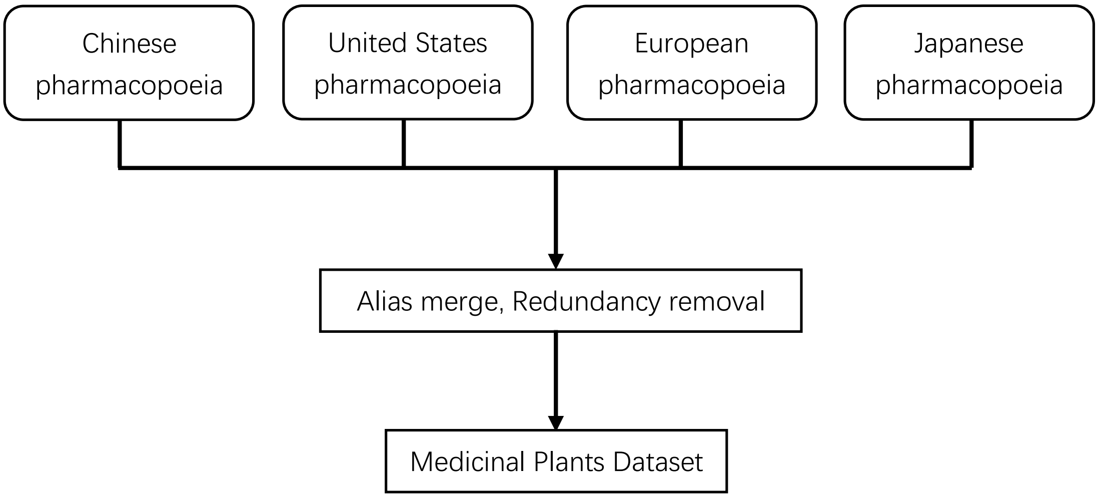

Figure A. Flowchart of medicinal plants data collection.

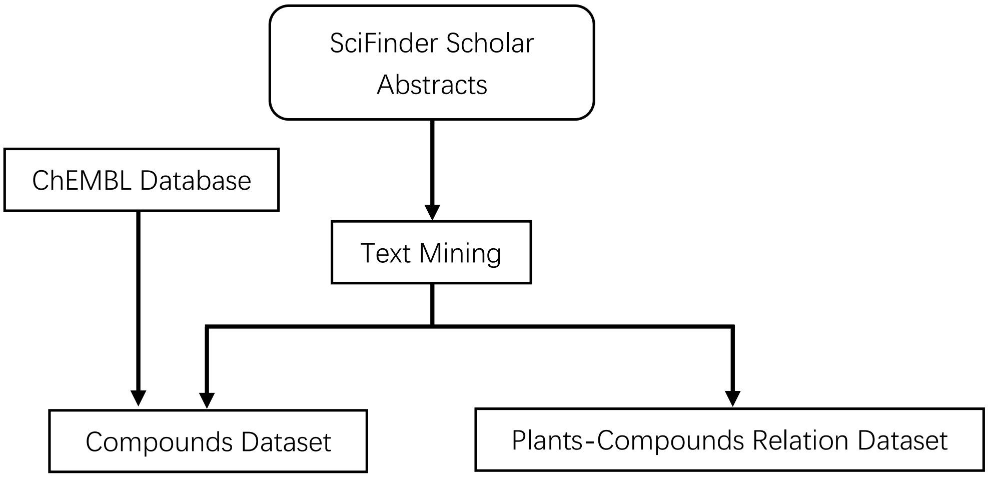

Figure B. Flowchart of compounds data, plants-compounds relation dataset collection

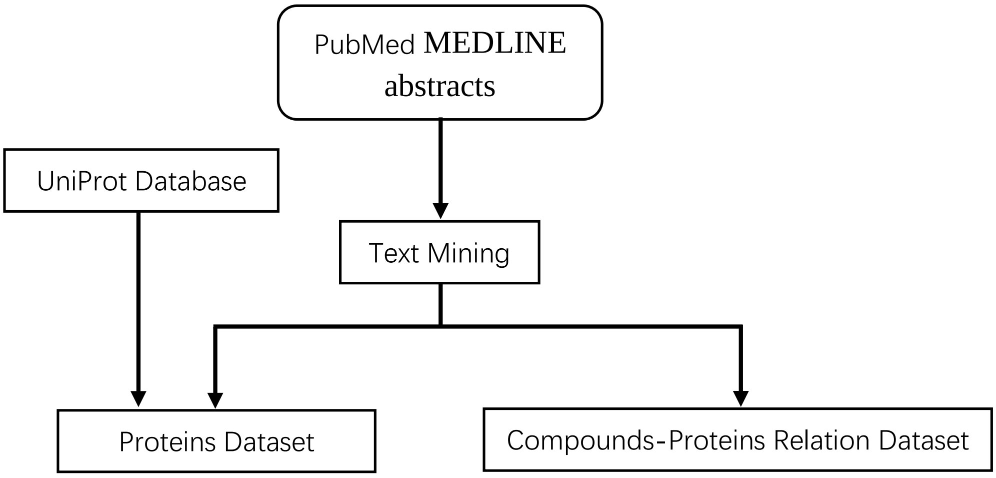

Figure C. Flowchart of proteins data, compounds-proteins relation dataset collection
