# Supplementary material for: TarNet: An Evidence-Based Database for Natural Medicine Research
Source: PLoS One. 2016 Jun 23;11(6):e0157222. doi: 10.1371/journal.pone.0157222 (PMC4919029; doi:10.1371/journal.pone.0157222)
Supplement: S1 Table — (PDF) [file pone.0157222.s002.pdf]

Table. The comparison with other similar databases

	**PPI data**	**Network Export**	**Data upload**	**Disease query**	**Topological analysis**	**Formula/Herbs query**	**Data Source**	**Substructure search**
TarNet	S*	S	S	NS	S	Herbs	Literatures and databases	S
TCMSP	NS*	NS	NS	S	Degree	Single herb	Databases	NS
TCMID	NS	NS	NS	S	NS	Formula or Single herb	Literatures and Databases	NS
BATMANTCM	NS	NS	NS	NS	NS	Formula or Single herb	Databases	NS
ASDB	NS	NS	NS	NS	NS	NS	Databases	S

*: S - Support; NS - Not Support
